# Genomics, Multi-Omics, and Emerging Artificial Intelligence for Combined Drought–Heat Stress Resilience in Plants: A Structured Narrative Review

**DOI:** 10.3390/plants15162516

**Published:** 2026-08-20

**Authors:** Congshan Xu, Ruirui Chen, Weilong Li, Lulu Song, Shunqi Huang, Fuyuan Zhang, Yujun Liu, Xiaodong Huang, Nankai Li, Liji Du, Shuanghong Shen

**Affiliations:** 1 Anhui Science and Technology Achievement Transformation Promotion Center, Anhui Provincial Institute of Science and Technology, Hefei 230001, China; xucongshan1027@163.com (C.X.); 13856920744@163.com (R.C.); 18326652324@163.com (L.S.); huangshunqi2016@163.com (S.H.); ahskjtaizh@163.com (F.Z.); jingcao1981@126.com (Y.L.); huangxiaodong1996@163.com (X.H.); zhenzhidashu66@163.com (N.L.); leonardo1317@163.com (L.D.); 2 Department of Artificial Intelligence, Anhui Zhongao Institute, Hefei 230001, China; 3 Institute of Artificial Intelligence, Hefei Comprehensive National Science Center, Hefei 230031, China

**Keywords:** combined drought–heat stress, genomics, multi-omics, transcriptomics, metabolomics, epigenomics, genomic prediction, machine learning, crop resilience, recovery

## Abstract

Combined drought–heat stress is not adequately described by adding the responses to drought and heat in isolation. Water limitation restricts evaporative cooling, whereas high temperature increases atmospheric demand and threatens photosynthesis, proteostasis, and reproduction. To assess what has been demonstrated rather than merely proposed, we combined a structured narrative review with a study-level evidence map spanning genomics, multi-omics, and emerging artificial intelligence (AI). The PubMed search, updated on 9 August 2026, returned 254 records; targeted and backward searches contributed eight seminal or reviewer-nominated publications. Independent screening retained 90 verified publications: 69 direct combined-stress studies, 11 genomics studies, six original AI or prediction studies, and four reviews or quantitative syntheses used for context. No conserved molecular signature emerged. Response direction varied with soil water status, vapor-pressure deficit, stress order, tissue, and developmental stage. Carbon allocation, redox and proteostasis balance, reproductive failure, and recovery recurred across omics layers, although neither their direction nor their adaptive value was consistent. Genomic loci and predictive performance were also environment dependent. Direct uses of AI remain uncommon and currently support prioritization more convincingly than causal inference. We organize this uneven evidence as a ladder extending from molecular association to functional validation, multi-environment prediction, and cultivar deployment. The main shortage is not another nominal omics layer but harmonized factorial experiments that measure recovery and reproduction, report field-relevant environmental metadata, and include external validation.

## 1. Introduction

Drought and heat now overlap during crop development often enough that their combination warrants study in its own right. Across crops, the joint treatment commonly reduces yield more than either component stress, although the size of the loss varies with species, developmental stage, treatment intensity, and experimental environment [1]. Early experiments in tobacco and Arabidopsis supplied a clue to the underlying problem: concurrent drought and heat produced transcript and metabolite patterns that were absent from the component treatments [2,3]. Later factorial studies did not reveal one uniform combined state. Depending on the endpoint and context, the response can be drought-dominant, heat-dominant, additive, antagonistic, or specific to the combination [4,5,6,7,8].

Part of this variability is physiological. Progressive soil drying tends to favor stomatal closure and hydraulic protection, yet heat dissipation may require continued transpiration. The apparent conflict is useful, but it is not a binary switch. Leaf water potential, VPD, hydraulic conductance, boundary-layer properties, treatment duration, and genotype all influence the outcome. Tomato cultivars differed in both leaf and flower cooling through stomatal behavior and ABA metabolism [9]. Field-grown soybean also showed genotype-dependent transpiration under combined water deficit and heat [10]. Even when stomata are considered, mesophyll conductance may impose a further photosynthetic limitation [11]. For this reason, soil water deficit and atmospheric drought caused by high VPD cannot be treated as interchangeable experimental conditions.

No single data layer resolves this context. QTL mapping and GWAS identify loci whose effects change among well-watered, drought, heat, and combined environments [12,13,14]. Transcript data capture regulatory state, whereas proteins, phosphorylation, and metabolites reveal different temporal and functional consequences. Deep phenotyping is needed to connect those signals to plant performance [15,16,17,18]. AI and ML offer ways to integrate the resulting data, but a successful prediction is not, by itself, a mechanism. Direct combined-stress applications remain much less common than general AI-in-breeding frameworks [19,20].

Three recent reviews set the background for this topic: Xu et al. organized the literature from an omics perspective [21]; Li et al. compared drought, heat, and combined treatments [22]; and an AI-augmented multi-omics review examined hormone systems across abiotic stresses [23]. Our question is narrower. We map each publication by evidence type and validation stage, separating direct combined-stress findings from extrapolations based on a single stress. We also examine whether the experimental design supports an additive or interactive interpretation, or only shows that a feature is specific to the combination. The biological scope remains combined drought–heat stress. Experimental principles may transfer to other stress combinations, but molecular conclusions require their own evidence.

## 2. Literature Search and Evidence Assessment

### 2.1. Review Type and Search Strategy

**Figure 1:**
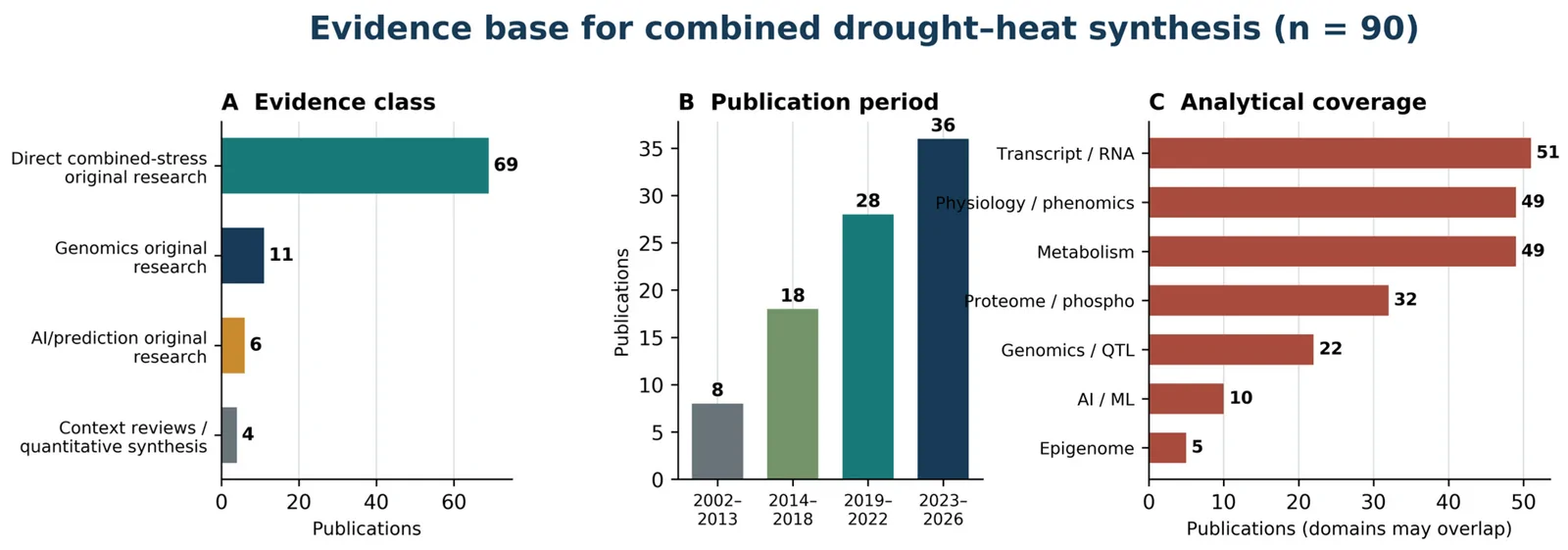
Quantitative evidence map of the 90 verified publications by evidence class, period, and analytical domain.

**Figure 2:**
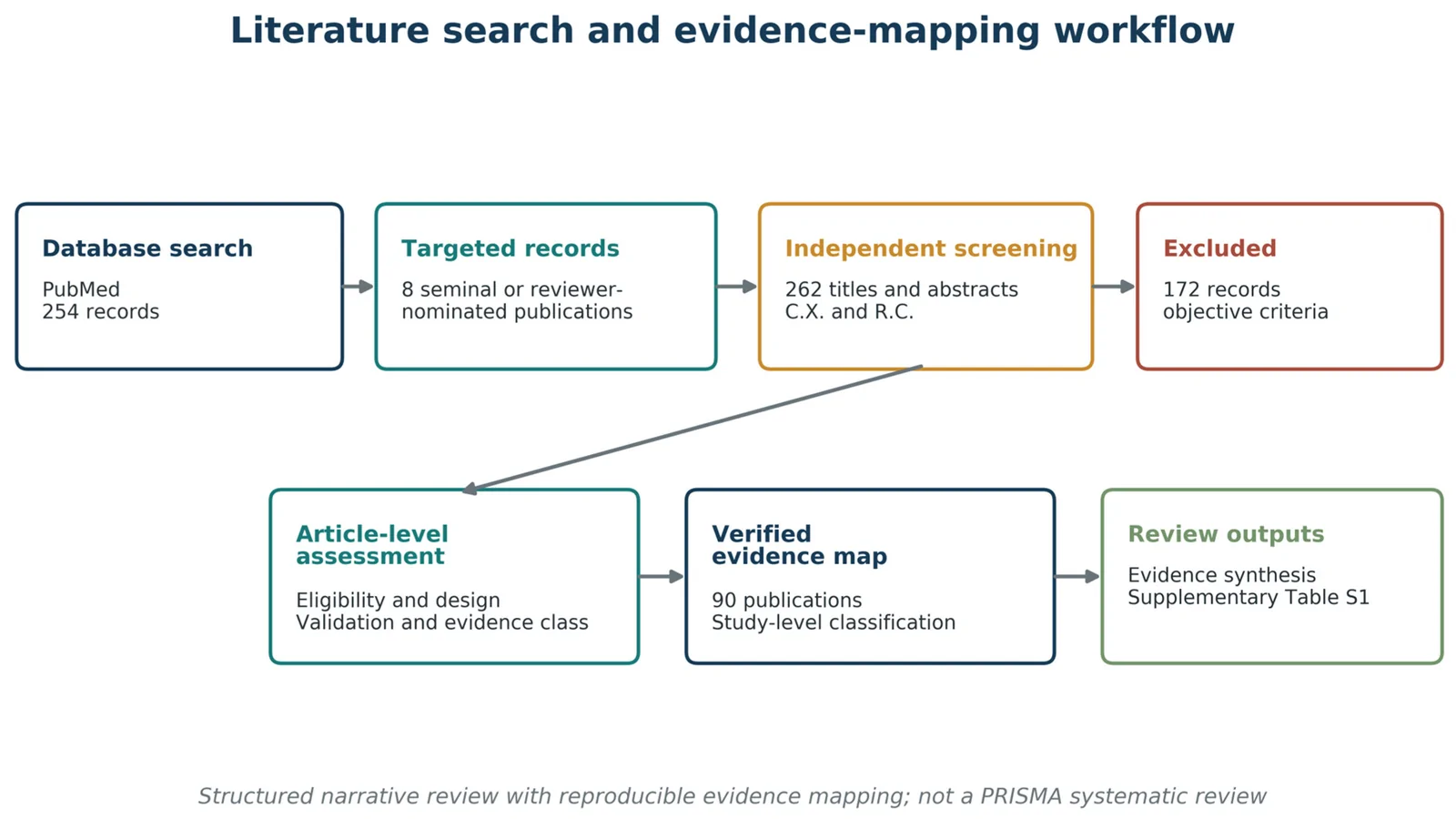
Structured literature identification, screening, classification, and evidence-assessment workflow.

This article is a structured narrative review with reproducible evidence mapping, not a systematic review or meta-analysis. The design was selected because the eligible literature is heterogeneous in species, stress delivery, developmental stage, molecular layer, and endpoint, making a pooled effect estimate inappropriate (Figure 1). PubMed was searched from database inception to 9 August 2026 using the full query provided in Supplementary File S1. The search combined terms for drought; heat or high temperature; simultaneous or sequential exposure; plants or major crops; and genomics, transcriptomics, proteomics, metabolomics, epigenomics, phenomics, GWAS, QTL, genomic prediction, ML, or AI. The search returned 254 records. Eight additional records were added through backward searching and targeted retrieval of seminal or reviewer-nominated studies not captured by the title/abstract query, producing 262 candidate records. The numerical flow is internally reconciled: 254 database records plus eight targeted records yielded 262 candidates; 172 were excluded during screening or article-level assessment, leaving 90 publications for study-level evidence mapping (Figure 2).

C.X. and R.C. independently screened titles and abstracts. W.L. resolved disagreements. Eligible publications met at least one objective criterion: (i) an original plant experiment containing a combined drought–heat treatment; (ii) a genomics, phenomics, or prediction study evaluating drought, heat, and/or their combination in a design informative for combined-stress selection; (iii) an original AI/ML study directly relevant to stress-response prediction or multimodal prioritization; or (iv) a review or quantitative synthesis required to position novelty or aggregate yield effects. Records were excluded when they lacked plant data, did not address drought or heat, reported only generic stress detection without molecular or breeding relevance, duplicated another report, or provided no extractable experimental information.

The reviewers extracted publication metadata, species, tissue or developmental stage, stress sequence, data layers, analytical method, validation design, major result, and evidence relationship. Disagreements in classification were reconciled by discussion. Ninety publications were retained in the evidence map: 69 direct combined-stress original studies, 11 genomics original studies, six AI/prediction original studies, and four contextual reviews or quantitative syntheses. Supplementary Table S1 classifies every included publication. No protocol was registered, and the workflow should not be interpreted as PRISMA-compliant systematic review methodology.

### 2.2. Definitions Used for Evidence Interpretation

The term combined drought–heat stress refers here to experiments in which water limitation and supra-optimal temperature overlap for at least part of the treatment. Simultaneous exposure, drought followed by heat, heat followed by drought, progressive drying combined with a heatwave, and recovery after treatment are retained as separate designs. PEG-induced osmotic stress is not assumed to reproduce soil drying because it differs in hydraulic and root-zone properties.

We use “additive” only when the joint response is consistent with an explicit null expectation on the scale analyzed. “Synergistic” and “antagonistic” require a statistically supported drought × heat interaction that is more or less extreme than that expectation. “Dominant” denotes a combined response resembling one component stress. “Combination-specific” denotes a feature detected only, reversed, or differently regulated under the combination; it does not automatically imply synergy. When a paper had not reported a factorial interaction test, we describe contrasts rather than infer non-additivity. Tolerance refers to performance under stress, acclimation to adjustment during exposure, recovery to restoration after stress removal, and resilience to maintenance and/or recovery of function. Molecular response alone is not treated as evidence of resilience (Table 1).

## 3. Experimental Context Determines the Combined-Stress State

### 3.1. Water Supply, Atmospheric Demand, and Stomatal Cooling

The often-cited conflict between drought-induced closure and heat-induced opening is a useful starting hypothesis but an incomplete mechanism. Soil water deficit lowers hydraulic supply; high VPD raises atmospheric demand; and leaf temperature emerges from their interaction with radiation, canopy architecture, and conductance. Under mild soil drying, some genotypes maintain enough transpiration to cool leaves, whereas severe drying can force closure regardless of temperature. Wheat tolerance under combined stress has been associated with maintained transpiration and water-soluble carbohydrates in grain [24]. In tomato, ABA metabolism and stomatal responses differed between leaves and flowers, demonstrating that canopy-average measurements can miss reproductive thermal exposure [9]. In chestnut and soybean, drought could dominate physiological impairment while heat altered the magnitude or route of adjustment [10]. These findings argue against a universal “open versus closed” model.

Molecular readouts cannot be separated from this physical context. ABA accumulation, aquaporin regulation, guard-cell signaling, and heat-shock responses need to be interpreted together with soil moisture, VPD, leaf temperature, and hydraulic traits. Otherwise, a transcript or metabolite assigned to a unique combined-stress state may simply reflect a difference in tissue temperature or dehydration. At minimum, experiments should report substrate water status, air temperature, relative humidity or VPD, radiation, stress ramp rate, leaf temperature, and treatment duration. The distinction is consequential, where elevated CO_2_ improved physiological performance in spring wheat exposed to anthesis-stage heat and drought, yet final yield was not preserved [25]. Short-term physiological protection was therefore not equivalent to agronomic resilience.

### 3.2. Stress Order, Intensity, and Recovery Are Biological Variables

Stress chronology changes the state from which the second perturbation is encountered. Progressive drought before a heatwave can precondition osmotic adjustment and antioxidant capacity but reduce cooling; heat before drought can accelerate water loss and alter subsequent ABA sensitivity. Arabidopsis exposed to combined and sequential stresses showed distinct acclimation trajectories [26], while rice genotypes displayed treatment-severity- and recovery-dependent transcriptional dynamics [27]. Earlier transcriptome–metabolome co-analysis also showed that humidity and stress order altered apparent heat–drought relationships [28]. Consequently, simultaneous and sequential treatments should not be pooled without explicit stratification. A rice experiment combining drought, salinity, and extreme temperature [29] and an Arabidopsis heat–drought–virus factorial experiment [30] further show that evidence from higher-order stress combinations should not be conflated with direct drought–heat effects.

Recovery is not simply the inverse of stress entry. Rice source and sink organs followed different metabolic trajectories after the combined stress in the field [31], and nutrient status modified both injury and recovery in another factorial study [32]. Persistent carbon depletion, reproductive damage, or proteome oxidation can outlast normalization of leaf water status. Sampling only at peak stress therefore favors signaling pathways while underrepresenting repair, remobilization, and yield determination. Recovery-focused work in Cycas likewise linked rehydration to structural and transcriptional repair of the photosynthetic system [33].

### 3.3. Developmental and Tissue Specificity

Reproductive tissues are disproportionately vulnerable because pollen development, fertilization, and grain filling have narrow thermal and carbon margins. Joint transcriptomic and metabolomic analysis of rice floral organs linked reproductive failure to sugar starvation [34]; maize pollen studies implicated sucrose synthase and associated metabolic remodeling in viability under heat and drought [35]. Soybean reproductive tissues showed stress- and tissue-specific transcriptional responses [36], and recent rice source–sink work connected sucrose transport with pollen germination and spikelet fertility [37]. These studies provide stronger yield-proximal evidence than vegetative marker induction. A recent flowering-stage soybean study further connected combined stress with coordinated physiological, biochemical, and transcriptomic changes [38].

Roots also require direct sampling. Maize primary-root responses to combined drought and heat involved salicylic-acid metabolism [39], but root molecular data remain less common than leaf transcriptomes. The imbalance matters because root hydraulic conductance, soil exploration, and root-to-shoot signaling condition leaf temperature and carbon status. A credible systems model therefore needs at least one hydraulic or root endpoint and one reproductive or yield endpoint, not only leaf omics.

## 4. Genomics: Environment-Dependent Architecture Rather than Universal Tolerance Loci

### 4.1. QTL and GWAS Evidence

Combined-stress genomics has advanced beyond candidate-gene lists. In maize, a panel of 300 inbred lines genotyped with 381,165 SNPs was evaluated across well-watered, drought, heat, and combined drought–heat environments; GWAS and genomic prediction showed that grain-yield and flowering architecture changed across treatments [12]. Independent tropical maize GWAS identified loci for yield under terminal drought and combined heat–drought conditions [40]. Wheat studies have mapped QTL or marker associations for adaptive traits, yield components, stay-green, stem reserve mobilization, and pre-anthesis combined stress [41,42,43,44,45]. More recent wheat panels extended this evidence to 345 genotypes under greenhouse and field heat–drought environments and to Aegilops-tauschii-derived kernel traits [46,47].

Across these studies, marker effects changed with population, phenology, environment, and trait definition. Some loci were detected under both heat and combined stress, whereas others appeared only in one environment. This pattern offers little support for a universal combined-stress tolerance locus. It instead places genotype × environment interaction and maturity confounding at the center of the analysis. Associations from all four factorial environments—particularly those designed to limit agronomic confounding—consequently carry more interpretive weight than results from a single severe treatment.

### 4.2. From Association to Validated Alleles

Translation improves when association is followed by genetic confirmation. A wheat study evaluated 315 accessions and used near-isogenic material to validate novel alleles associated with combined drought–heat tolerance [48]. Transcripts at a chromosome 6B locus were associated with yield, leaf mass, and chlorophyll under combined stress [49]. These examples move beyond expression correlation, but they still do not constitute cultivar deployment. The evidence ladder should distinguish: locus discovery; fine mapping or causal-variant resolution; functional validation; introgression or pre-breeding; multi-environment testing; and released-cultivar performance.

Structural variation and pangenomes could reveal adaptive alleles that a single reference genome misses, especially in polyploid crops. However, direct evidence linked to combined drought–heat performance remains scarce in the mapped literature. Pangenomics is therefore an enabling strategy here and is not yet a demonstrated combined-stress result. A practical next step would pair haplotype-resolved panels with factorial phenotypes, then test promoter or copy-number variants at validated loci across environments.

### 4.3. Genomic and Phenomic Prediction

A useful prediction must survive the environmental structure of the breeding problem. In maize, hyperspectral and marker data were integrated to estimate physiological genomic breeding values for grain yield across combined heat–drought environments [19]. High-throughput phenotyping has also aided genetic dissection in maize and durum wheat [50,51], and nondestructive phenomics predicted anthesis-stage tolerance in Brassica [52]. Secondary traits can thus improve selection, but reported accuracy depends strongly on how validation is arranged. Random cross-validation is liable to optimism when related genotypes, or observations from the same environment, occur in both training and test sets. Leave-environment-out and leave-genotype-out tests better approximate deployment (Table 2).

## 5. Multi-Omics Reveals Conditional Convergence and Layer-Specific Disagreement

### 5.1. Transcriptomes: Interaction, Dominance, and Alternative Splicing

Transcriptomics dominates the direct evidence, which also makes it the easiest layer to overgeneralize. Studies in Arabidopsis, sorghum, wheat, soybean, lentil, switchgrass, grapevine, coffee, and potato compared component and combined treatments [6,7,8,18,53,54,55,56,57,58]. Some found transcripts confined to combinations; others found profiles dominated by drought or heat. The apparent disagreement becomes less surprising once treatment intensity, tissue, and sampling time are considered. Even then, a combination-specific transcript indicates regulatory interaction only when supported by a factorial contrast, and induction alone says nothing about adaptive value. Stage-resolved wheat data with functional characterization of TaEREBP1-L, together with paired transcriptome–metabolome profiling in date palm, illustrate how developmental stage and analytical layer alter the interpretation [59,60].

Post-transcriptional regulation adds another dimension. Wheat and tea studies documented extensive alternative splicing under drought, heat, and their combination [61,62,63]. Combined-stress regulation may therefore change isoform usage without large gene-level abundance shifts. Co-expression and cis-regulatory analyses have identified candidate promoter logic [64], but motif enrichment remains associative until promoter editing or reporter assays test function under the combined treatment.

### 5.2. Proteins, Phosphorylation, Redox State, and Proteostasis

Protein abundance often agrees only partly with transcript abundance because translation, turnover, compartmentation, and damage operate on different timescales. Comparative proteomics in barley, maize, wild barley, Carissa, potato, and Marchantia identified stress- and genotype-dependent remodeling [65,66,67,68,69,70,71]. Recurrent functional categories include photosynthesis, chaperones, antioxidant systems, central metabolism, and protein turnover, but individual proteins are not consistently regulated across species. A leaf–root sorghum proteomic dataset now adds tissue-resolved evidence from contrasting genotypes [72].

Maize phosphoproteomics provides the clearest direct view of rapid signaling: phosphorylation changed under drought, heat, and their combination [15]. The result is consistent with kinase-centered regulation, but one small evidence base cannot establish a universal ROS–calcium–kinase hub. Calcium-dependent kinases, ROS signaling, and MAPK cascades remain plausible organizing mechanisms. Demonstrating their recurrence and causal order across species will require targeted phosphoproteomics, kinase perturbation, and time-resolved experiments.

Proteostasis is similarly conditional. Heat-shock factors and chaperones are expected contributors, but several frequently cited wheat and Arabidopsis HSF studies validate drought responses rather than combined drought–heat performance. They should be treated as candidate links between ABA, redox control, and proteostasis, not as established combined-stress master regulators. Direct combined-treatment gene editing or allele substitution is still needed. In citrus subjected to drought, heat, and high irradiance, tolerance was associated with lower heat pressure and activation of photosystem II repair [73]. OsMYB55 overexpression in maize improved performance under individual and combined heat–drought treatments [74], whereas Arabidopsis APX1 perturbation provided direct functional evidence for redox control under the combination [75].

### 5.3. Metabolism Connects Molecular State to Function

Metabolomics provides a closer readout of carbon balance, osmotic adjustment, and oxidative cost. Rice field studies showed cultivar-, organ-, and recovery-specific metabolic responses [16,31]. Arabidopsis experiments under ambient and elevated CO_2_ demonstrated that metabolic trajectories change across repeated combined-stress periods [76]. Citrus, grapevine, cotton, and Eucalyptus studies likewise indicate that a protective versus damaging metabolism depends on the genotype and context [4,77,78,79]. In Brachypodium, integrated physiological, metabolic, and transcriptional analysis linked drought–heat combinations to life-history trade-offs between escape and defense [80].

Across these systems, sugars, amino acids, organic acids, antioxidants, and secondary metabolites recur, but the direction is not uniform. Soluble sugars can function as osmoprotectants and signals, yet their accumulation may also indicate impaired sink use. Antioxidant induction can represent successful buffering or a response to greater damage. Interpretation therefore requires flux, tissue context, and performance endpoints rather than metabolite abundance alone.

### 5.4. Integrative Studies: What Is Genuinely Added?

Placing several omics lists side by side is not, by itself, integration. The stronger studies changed the biological inference: a wheat atlas connected transcript, protein, metabolite, and phenotype modules across combinatorial treatments [17], while potato multi-omics with deep phenotyping resolved genotype- and treatment-specific trajectories that no single layer recovered [18]. Arabidopsis holobiont analyses linked host metabolism with microbial-community change [81,82], and rice floral and maize pollen datasets connected molecular remodeling to fertility [34,35]. Together, these studies point to conditional convergence in carbon allocation, proteostasis and redox balance, reproductive function, and recovery. Integrated analyses in Dendrobium and citrus add support, although the citrus experiment also included high irradiance [83,84].

Cross-layer disagreement is equally informative. Transcripts may respond before proteins, metabolites may reflect injury rather than regulation, and a highly connected network node need not be causal. An integrative analysis should therefore report concordance and discordance rather than forcing all layers into one consensus network. Sampling time also matters: early signaling, sustained acclimation, damage, and recovery are different biological states.

### 5.5. Epigenetics and Non-Coding RNA: Direct Evidence Is Emerging

**Figure 3:**
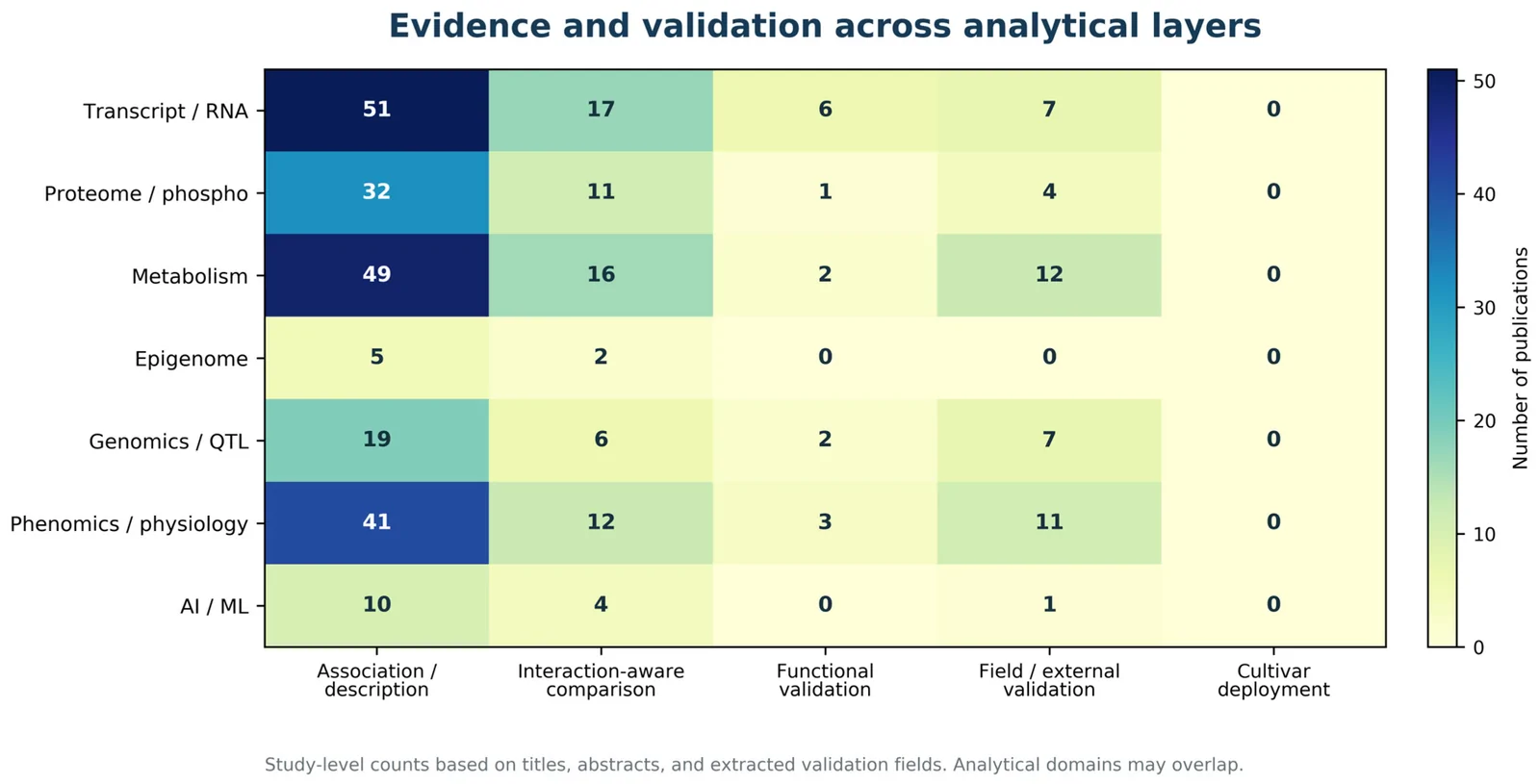
Evidence matrix linking experimental context, omics layer, supported inference, and validation ceiling.

Direct combined-stress epigenomic evidence is beginning to appear. Switchgrass H3K4me3 profiling identified chromatin states across drought, heat, and their combination [85]; maize work linked DNA methylation, expression reprogramming, and flowering under combined stress [86]; and barley studies have begun mapping genetic and epigenetic stress memory [87]. Potato microRNA profiles also differed between contrasting cultivars under combined drought and heat [88] (Table 3). These findings justify an emerging research area, not a settled universal mechanism. DNA methylation, histone marks, lncRNAs, and microRNAs should be tested for persistence, inheritance, and functional relevance using matched genetic backgrounds and recovery or transgenerational designs (Figure 3).

## 6. Emerging AI: Useful for Prioritization, Not a Substitute for Causal Validation

### 6.1. Distinguishing Analytical Terms

The analytical labels used in this literature are not interchangeable. Machine learning (ML) learns predictive relationships from data; deep learning uses multi-layer neural networks; graph learning represents relations among genes, pathways, genotypes, or environments; and conventional genomic prediction estimates breeding values from markers and relatedness. Each answers a different question. An image model may classify stress accurately without identifying a tolerance mechanism, just as predictive accuracy alone cannot establish causality.

Only a few original studies apply these methods directly to combined stress. One artificial neural network assembled plant experiments to predict combined-stress impact and interaction, reporting 76.33% overall accuracy and a rice-yield check [20]. It is a useful proof of concept, although heterogeneity among source experiments and limited external biological validation restrict mechanistic interpretation. Physiological genomic prediction in maize combined hyperspectral and marker data for yield under heat and drought [19]. A rice ML study linked genomic features to heat- and drought-responsive transcription [89], which is relevant to regulatory prioritization but is not a direct causal test of the combination. Interpretable ML has also guided follow-up nanomaterial seed-treatment experiments [90]. This closed prediction–experimentation loop is promising, even though its present evidence is indirect to drought–heat stress.

### 6.2. What AI Can Add Now

The near-term value of AI lies in narrowing an experimental decision. Genotype, expression, network position, and prior annotation can be combined to rank targets for perturbation; markers, thermal or hyperspectral traits, weather, and yield can be linked across environments in a multimodal model. Active learning adds a more practical question: which genotype × environment × sampling-time observation would reduce uncertainty most? Until those ranked outputs are tested, they remain hypotheses rather than mechanisms.

Interpretability needs to be tied to a method and a decision. SHAP values or permutation importance rank features, saliency maps locate image regions, biologically constrained networks restrict implausible edges, and counterfactual analysis asks which input changes would alter a prediction. These outputs are not interchangeable, and attribution can shift with correlated inputs or the chosen background distribution. A large SHAP value for a gene or metabolite is therefore a reason to test it, not evidence that its perturbation will improve tolerance.

### 6.3. Failure Modes in Sparse Compound-Stress Data

Sparse combined-stress datasets magnify familiar modeling failures. Samples from the same genotype, experiment, or time series can leak across training and test sets; laboratory or sequencing batch can become a surrogate for stress class; and incomplete modalities may reduce analysis to the smallest fully observed subset. A separate problem appears at deployment. Chamber-trained models encounter domain shift when they are applied to field heatwaves or unfamiliar germplasm. Class imbalance and overfitting compound each of these risks (Table 4).

At minimum, reports should state the biological-unit sample size, feature count, preprocessing, treatment of missing data, baseline models, hyperparameter selection, split unit, uncertainty, external validation, and code or data availability. Breeding applications are better tested by leaving out families, genotypes, or environments than by randomly splitting observations. For mechanistic prioritization, the standard should be more demanding: at least one predicted regulator should be perturbed under the combined treatment.

## 7. From Molecular Association to Crop Improvement

Calling every stress-responsive gene ‘breeding relevant’ compresses several distinct stages into one claim. Candidate discovery establishes correlation or network priority; functional validation asks whether perturbation changes a phenotype; pre-breeding moves an allele into useful germplasm; and genomic prediction ranks material in a defined target population. Stability is then tested across environments. Deployment comes later and requires agronomic value with acceptable trade-offs. Evidence should be labeled by the highest stage actually completed.

The best-supported targets are traits and alleles linked to performance under direct combined treatment. Wheat near-isogenic validation [48], field GWAS and QTL studies [14,43,44], maize genomic/phenomic prediction [12,19], and reproductive carbon-allocation mechanisms [34,35,37] can provide starting points. However, the evidence map identified no broadly deployed cultivar whose release was attributed to a genomics–multi-omics–AI pipeline for combined drought–heat stress. This absence should temper translational claims.

**Figure 4:**
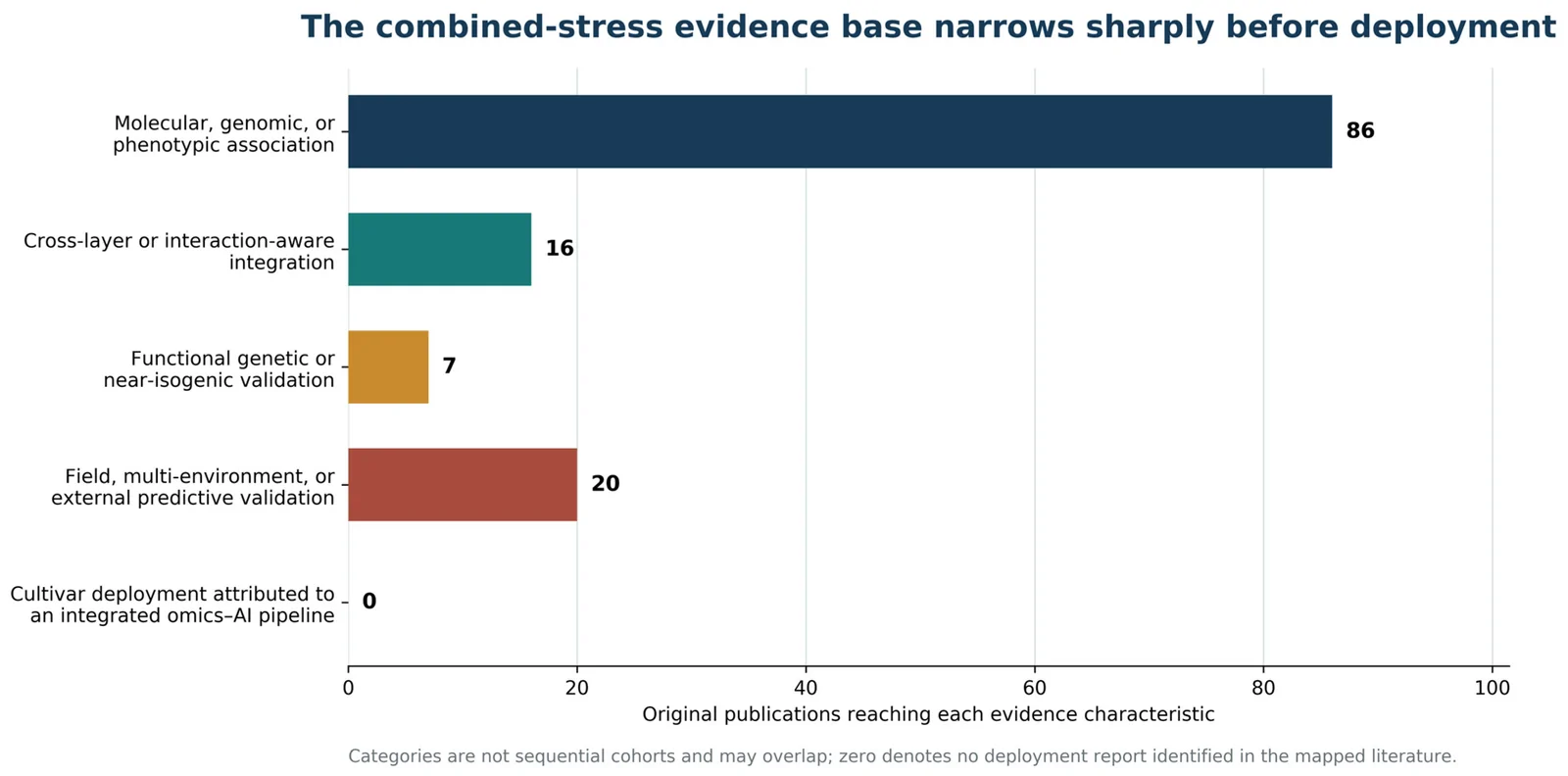
Evidence ladder from combined-stress association to cultivar deployment, showing the current concentration of evidence at discovery and the validation bottleneck.

The pipeline should begin with a factorial target-product profile that specifies the crop stage, drought trajectory, heatwave pattern, VPD, and yield endpoint. Then, genomics defines segregating variation; multi-omics indicates when and where alleles act; and phenomics measures cooling, hydraulics, fertility, and recovery. AI can prioritize crosses, environments, or perturbations, but it does not close the loop. Closure requires predicted alleles or modules to improve yield stability across target environments without an unacceptable penalty under favorable conditions (Figure 4).

## 8. Research Gaps and Priorities

Environmental realism and comparability must be improved together. Soil water status, VPD, tissue temperature, stress ramp, chronology, developmental stage, and recovery are often reported unevenly, which makes ostensibly similar experiments difficult to compare. Control, drought, heat, and combined treatments provide the minimum factorial contrast for estimating interaction. Reciprocal sequential treatments address another biological question and should remain analytically separate (Table 5).

Current sampling is still dominated by vegetative leaf transcriptomes. Roots, guard cells, vasculature, pollen, ovules, developing grain, and source–sink interfaces are harder to collect, yet these tissues often determine hydraulic failure or yield loss. The same imbalance affects phosphorylation, protein turnover, chromatin accessibility, and flux-informed metabolism. Recovery studies should follow restoration of function, fertility, or yield rather than stop at a transcript measured shortly after rewatering.

A clearer route from association to causality is also needed. Candidate HSFs, kinase modules, epigenetic marks, splice isoforms, and network hubs can be examined by allele replacement, promoter editing, inducible perturbation, or tissue-specific manipulation. Crucially, the test must retain the factorial combined treatment and measure trade-offs in cooling, water use, phenology, and yield. Without that step, a responsive candidate remains only a candidate.

Many AI benchmarks bear little resemblance to deployment. Shared datasets need genotype identity, complete environmental metadata, raw and processed molecular data, and prespecified train/test partitions. Linear mixed models and conventional genomic prediction should remain visible baselines rather than weak comparators omitted in favor of complex models. Performance on unseen genotypes and environments, followed by a prospective test, matters more than a small gain under random cross-validation. Transfer learning may help when samples are scarce, but only if domain shift is measured rather than assumed away.

Several design principles extend beyond drought and heat. Factorial null models, explicit chronology, cross-layer concordance, and validation ladders are also useful for salinity–heat, flooding–heat, nutrient–drought, and biotic–abiotic combinations. The molecular state is less portable. It has to be demonstrated in each stress context.

## 9. Conclusions

Combined drought–heat stress is better understood as a context-dependent state than as a conserved pathway. Direct studies document changes in transcription, splicing, phosphorylation, metabolism, chromatin, physiology, and reproduction, but the direction and adaptive value of those changes vary with water supply, atmospheric demand, stress order, tissue, and genotype. The most consistent points of convergence are carbon allocation, proteostasis and redox balance, reproductive function, and recovery. Even these are conditional. A universal ABA–HSF, ROS–calcium–kinase, or epigenetic control system has not been demonstrated.

Genomics has uncovered environment-dependent loci and enabled prediction under combined stress. Integrated omics has been most useful where it exposes agreement and disagreement among molecular layers, while AI has begun to prioritize interactions and connect genomic with phenomic information. Its present reach is limited by sparse datasets and weak external validation. In our assessment, progress now depends less on adding candidate lists than on testing them in harmonized factorial experiments that include reproductive and recovery phenotypes, causal perturbation, and prospective field validation. That evidence is still the missing link between molecular association and crop improvement.

## Figures and Tables

**Table 1:** Operational definitions and minimum evidence required in this review.

Term	Operational Meaning	Minimum Evidence	Common Overinterpretation Avoided
Additive	Joint effect matches a stated null expectation	Factorial model or explicit additivity rule on the analyzed scale	Calling any intermediate phenotype additive
Synergistic	Joint effect exceeds the null expectation	Significant drought × heat interaction and directionally greater effect	Equating greater injury than single stress with synergy
Antagonistic	Joint effect is smaller than the null expectation	Significant interaction and attenuated joint effect	Assuming one stress simply “protects” against the other
Dominant	Combined profile resembles one component treatment	Multivariate or trait-level similarity to drought or heat	Treating dominance as absence of interaction
Combination-specific	Feature occurs only, reverses, or changes rank under the combination	Factorial contrasts; interaction preferred	Treating uniqueness as proof of adaptive value
Tolerance	Relative performance during exposure	Biomass, fertility, photosynthesis, or yield compared under matched stress	Inferring tolerance from marker induction
Recovery	Functional restoration after stress removal	Post-stress time point(s) with physiological or yield endpoint	Inferring recovery from measurements made during stress
Resilience	Maintenance and/or recovery of function	Stress plus recovery or yield stability evidence	Using resilience as a synonym for molecular response

**Table 2:** Representative genomics and prediction studies informative for combined drought–heat resilience.

Study	Crop and Design	Genomic/ Phenomic Input	Endpoint and Validation	Evidence Contribution	Remaining Limitation
Yuan et al. [12]	Maize; 300 inbred lines; four water × temperature environments	381,165 SNPs	Yield and flowering; GWAS and cross-validation	Demonstrates environment-dependent architecture and prediction	External population validation needed
Schmidt et al. [48]	Wheat; 315 accessions plus near-isogenic material	Alleles and physiological traits	Combined-stress tolerance; genetic validation	Moves from association toward validated alleles	Multi-location deployment not shown
Thirunavukkarasu et al. [40]	Tropical maize germplasm	Genome-wide markers	Yield under terminal drought and combined heat–drought	Direct field GWAS	Population-specific effects
Tadesse et al. [14]	Bread wheat breeding material	Genome-wide markers	Heat and heat–drought resilience in field environments	Identifies stable and environment-specific regions	Causal variants unresolved
Qaseem et al. [43]	Bread wheat; association panel	SNPs and yield traits	Pre-anthesis combined stress in field conditions	Yield-proximal associations	Phenology confounding requires continued control
QTL study [44]	Wheat mapping population	Linkage markers	Yield component traits under combined stress	Defines genomic intervals	Fine mapping and validation required
Stay-green GWAS [45]	Wheat association panel	Markers; stay-green; reserve mobilization	Combined-stress yield-component traits	Links physiological traits with loci	Trait stability across sites unknown
Rutkoski et al. [19]	Tropical maize across environments	Markers plus hyperspectral bands	Grain-yield PGEBV	Direct multimodal prediction under combined stress	Validation remained within the study network

**Table 3:** Direct integrative and mechanism-oriented evidence under combined drought–heat stress.

Study	Species, Tissue, and Stage	Stress Design	Data Layers	Main Evidence-Based Finding	Interaction/ Validation Note
Rizhsky et al. [3]	Arabidopsis leaves	Concurrent drought and heat	Transcript and metabolite profiling	Established a combination-specific molecular state	Foundational factorial contrast
Da Ros et al. [17]	Wheat; contrasting genotypes	Combinatorial abiotic treatments	Transcriptome, proteome, metabolome, phenotype	Cross-layer modules differed among treatments and genotypes	Direct multi-omics; controlled design
Zagorščak et al. [18]	Potato; contrasting genotypes	Single and combined abiotic stress	Transcriptome, metabolome, hormones, deep phenotypes	Combined states reorganized molecular and phenotypic trajectories	Direct factorial integration
Senizza et al. [82]	Arabidopsis holobiont	Drought, heat, and their combination	Metabolomics, metabarcoding, physiology	Host and microbiome components changed jointly	Direct multi-omics; causality unresolved
Liu et al. [61]	Wheat	Drought, heat, and their combination	Transcriptome and isoform profiling	Alternative splicing added regulation beyond gene abundance	Direct treatment comparison
Hosseini et al. [57]	Lentil	Individual and combined treatments	Comparative transcriptomics	Genotype- and treatment-specific regulatory programs	Direct transcript evidence
Yang et al. [39]	Maize primary roots	Combined drought and heat	Root transcript/physiology and salicylic-acid analysis	Root response involved salicylic-acid metabolism	Mechanistic candidate; further genetics needed
Hu et al. [15]	Maize leaves	Factorial drought, heat, and combination	Phosphoproteomics and physiology	Combination altered phosphorylation beyond abundance data	Rare direct signaling-layer evidence
Lawas et al. [34]	Rice floral organs	Heat and drought during reproduction	Transcriptome and metabolome	Sugar starvation was associated with reproductive failure	Yield-proximal, tissue-resolved evidence
Maize pollen study [35]	Maize pollen	Heat and drought	Transcriptome, metabolome, functional assay	Sucrose synthase regulation was linked to pollen viability	Functional support at reproductive stage
Rice recovery studies [16,31]	Rice source and sink organs	Field combined stress and recovery	Metabolomics and physiology	Cultivar and organ trajectories persisted into recovery	Direct field and recovery evidence
Switchgrass chromatin study [85]	Switchgrass	Drought, heat, and their combination	H3K4me3 profiling	Stress-specific chromatin signatures were observed	Direct epigenomic association

**Table 4:** Original AI, ML, and multimodal prediction studies relevant to combined drought–heat research.

Study	Species/Sample	Input	Model/Task	Validation and Reported Performance	Interpretation Boundary
ANN combined-stress model [20]	Cross-study plant records; rice check	Environmental and stress descriptors	Artificial neural network predicting impact/interaction	Hold-out evaluation; 76.33% overall accuracy; rice yield comparison	Direct proof of concept, not causal mechanism; external scope limited
Physiological genomic prediction [19]	Tropical maize across environments	Markers plus full hyperspectral profiles	PGEBV for grain yield	Cross-environment evaluation within study	Direct combined-stress prediction; independent population needed
Maize GWAS/GP [12]	300 maize inbred lines	381,165 SNPs and four stress environments	Genomic prediction of yield and flowering	Cross-validation across WW, DS, HS, and DHS	Shows environment dependence; validation design governs portability
Rice transcription prediction [89]	Public rice expression datasets	Genomic/promoter features and expression labels	ML prediction of heat/drought-responsive transcription	Cross-validation with feature ranking	Supports prioritization; not a direct combined-stress experiment
Interpretable nanopriming ML [90]	Multi-crop nanopriming experiments	Material descriptors and stress phenotypes	Interpretable ML plus experimental selection	Cross-validation and follow-up experiments	Demonstrates closed-loop workflow; indirect to drought–heat combination
Brassica phenomic prediction [52]	Brassica at anthesis	Nondestructive phenomic traits	Prediction of heat/drought tolerance	Within-study trait prediction	Yield and external-environment validation remain necessary

**Table 5:** Minimum design and reporting priorities for the next generation of combined-stress studies.

Domain	Minimum Requirement	Stronger Design	Decision Enabled
Stress environment	Soil water status, temperature, humidity/VPD, duration	Tissue temperature, radiation, ramp rate, hydraulic measurements	Distinguish soil drought, atmospheric demand, and thermal exposure
Factorial inference	Control, drought, heat, combination with biological replication	Explicit drought × heat model across intensity or time	Additive, synergistic, antagonistic, dominant, or combination-specific classification
Chronology	State whether simultaneous or sequential	Reciprocal order plus recovery time course	Separate priming, interaction, damage, and recovery
Tissue and stage	Defined tissue and developmental stage	Root–shoot and reproductive source–sink sampling	Connect molecular state to fertility and yield
Multi-omics	Synchronized sampling and transparent integration	Cross-layer concordance, discordance, and perturbation	Identify regulatory bottlenecks rather than correlated lists
Genomics	Population, marker density, phenology, and G × E model	Fine mapping, haplotypes/SV, near-isogenic or edited validation	Move from locus association to causal allele
AI/ML	Biological sample size, baseline, split unit, metric	External genotype/environment test, uncertainty, prospective experiment	Assess portability and prioritize validation
Translation	Stress phenotype plus agronomic endpoint	Multi-environment yield stability and favorable-condition penalty	Distinguish candidate discovery from cultivar value

## Data Availability

No new experimental datasets were generated. The verified study-level evidence map and complete search string are provided in Supplementary File S1; bibliographic records are available through PubMed and the cited publishers.

## Supplementary Materials

The following supporting information can be downloaded at: https://www.mdpi.com/article/10.3390/plants15162516/s1, Supplementary File S1: Search Strategy and Study-Level Evidence Map. Table S1. Study-Level Evidence Map (n = 90).
