# Supplementary material for: Genomics, Multi-Omics, and Emerging Artificial Intelligence for Combined Drought–Heat Stress Resilience in Plants: A Structured Narrative Review
Source: Plants (Basel). 2026 Aug 20;15(16):2516. doi: 10.3390/plants15162516 (PMC13516375; doi:10.3390/plants15162516)
Supplement: Supplementary file 1 [file plants-15-02516-s001.zip › plants-4451217-supplementary.pdf]

# Supplementary File S1
# Search Strategy and Study-Level Evidence Map

## Full PubMed Search String

(drought[Title/Abstract] AND (heat[Title/Abstract] OR "high temperature"[Title/Abstract]) AND (combined[Title/Abstract] OR combination[Title/Abstract] OR simultaneous[Title/Abstract] OR concurrent[Title/Abstract])) AND (omics[Title/Abstract] OR genomic*[Title/Abstract] OR transcriptom*[Title/Abstract] OR proteom*[Title/Abstract] OR metabolom*[Title/Abstract] OR epigen*[Title/Abstract] OR phenom*[Title/Abstract] OR "machine learning"[Title/Abstract] OR "artificial intelligence"[Title/Abstract] OR "genomic prediction"[Title/Abstract] OR GWAS[Title/Abstract] OR QTL[Title/Abstract]) AND (plant*[Title/Abstract] OR crop*[Title/Abstract] OR Arabidopsis[Title/Abstract] OR wheat[Title/Abstract] OR maize[Title/Abstract] OR rice[Title/Abstract] OR soybean[Title/Abstract] OR potato[Title/Abstract]) AND ("2000/01/01"[Date - Publication] : "2026/08/09"[Date - Publication])

## Table S1. Study-Level Evidence Map (n = 90)

| Year | Study | Evidence class | Relationship | System / layers | Validation and interpretation | DOI |
|---|---|---|---|---|---|---|
| 2021 | Meta-analysis of drought and heat stress combination impact on crop yield and yield components. | review/context | context only | See article metadata/abstract | Original study; detailed design assessed in narrative where relevant | 10.1111/ppl.13203 |
| 2002 | The combined effect of drought stress and heat shock on gene expression in tobacco. | direct combined-stress original research | direct | See article metadata/abstract | Original study; detailed design assessed in narrative where relevant | 10.1104/pp.006858 |
| 2004 | When defense pathways collide. The response of Arabidopsis to a combination of drought and heat stress. | direct combined-stress original research | direct | See article metadata/abstract | Original study; detailed design assessed in narrative where relevant | 10.1104/pp.103.033431 |
| 2018 | Combined Drought and Heat Activates Protective Responses in Eucalyptus globulus That Are Not Activated When Subjected to Drought or Heat Stress Alone. | direct combined-stress original research | direct | See article metadata/abstract | Original study; detailed design assessed in narrative where relevant | 10.3389/fpls.2018.00819 |
| 2020 | Physiological, Biochemical, and Transcriptional Responses to Single and Combined Abiotic Stress in Stress-Tolerant and Stress-Sensitive Potato Genotypes. | direct combined-stress original research | direct | See article metadata/abstract | Original study; detailed design assessed in narrative where relevant | 10.3389/fpls.2020.00169 |
| 2022 | Global analysis of switchgrass (Panicum virgatum L.) transcriptomes in response to interactive effects of drought and heat stresses. | direct combined-stress original research | indirect/support ing | See article metadata/abstract | Original study; detailed design assessed in narrative where relevant | 10.1186/s12870-022-03477-0 |
| 2023 | Global transcriptome and gene co-expression network analyses reveal regulatory and non-additive effects of drought and heat stress in grapevine. | direct combined-stress original research | indirect/support ing | See article metadata/abstract | Original study; detailed design assessed in narrative where relevant | 10.3389/fpls.2023.1096225 |
| 2024 | Transcriptomic Analyses Reveal That Coffea arabica and Coffea canephora Have More Complex Responses under Combined Heat and Drought than under Individual Stressors. | direct combined-stress original research | direct | See article metadata/abstract | Original study; detailed design assessed in narrative where relevant | 10.3390/ijms25147995 |
| 2024 | Stomatal effects and ABA metabolism mediate differential regulation of leaf and flower cooling in tomato cultivars exposed to heat and drought stress. | direct combined-stress original research | indirect/support ing | See article metadata/abstract | Original study; detailed design assessed in narrative where relevant | 10.1093/jxb/erad498 |
| 2025 | Differential Transpiration of Soybean Plants Subjected to a Combination of Water Deficit and Heat Stress Under Field Conditions. | direct combined-stress original research | direct | See article metadata/abstract | Original study; detailed design assessed in narrative where relevant | 10.1111/ppl.70584 |
| 2024 | Mesophyll conductance limits photosynthesis in fluctuating light under combined drought and heat stresses. | direct combined-stress original research | direct | See article metadata/abstract | Original study; detailed design assessed in narrative where relevant | 10.1093/plphys/kiad605 |

| Year | Study | Evidence class | Relationship | System / layers | Validation and interpretation | DOI |
|---|---|---|---|---|---|---|
| 2018 | Genome-Wide Association Mapping and Genomic Prediction Analyses Reveal the Genetic Architecture of Grain Yield and Flowering Time Under Drought and Heat Stress Conditions in Maize. | AI/prediction original research | indirect/supporting | maize; 381,165 SNPs; flowering; yield | cross-validation across environments; factorial stress design | 10.3389/fpls.2018.01919 |
| 2018 | Genome-wide association mapping in bread wheat subjected to independent and combined high temperature and drought stress. | genomics original research | direct | bread wheat; SNPs; agronomic traits | field conditions; direct field evidence | 10.1371/journal.pone.0199121 |
| 2022 | Genomic analysis for heat and combined heat-drought resilience in bread wheat under field conditions. | genomics original research | direct | bread wheat; markers; field traits | field environments; direct field evidence | 10.1007/s00122-021-03969-x |
| 2015 | Phosphoproteomic analysis of the response of maize leaves to drought, heat and their combination stress. | direct combined-stress original research | direct | maize leaves; phosphoproteome; physiology | factorial controlled experiment; direct signaling-layer evidence | 10.3389/fpls.2015.00298 |
| 2019 | Metabolic responses of rice cultivars with different tolerance to combined drought and heat stress under field conditions. | direct combined-stress original research | direct | See article metadata/abstract | Original study; detailed design assessed in narrative where relevant | 10.1093/gigascience/giz050 |
| 2023 | Multi-omics atlas of combinatorial abiotic stress responses in wheat. | direct combined-stress original research | indirect/supporting | wheat; transcriptome; proteome; metabolome; phenotype | controlled multi-treatment experiment; direct integrative evidence | 10.1111/tpj.16332 |
| 2025 | Integration of multi-omics data and deep phenotyping provides insights into responses to single and combined abiotic stress in potato. | direct combined-stress original research | direct | potato; transcriptome; metabolome; hormones; deep phenotypes | controlled factorial experiment; direct integrative evidence | 10.1093/plphys/kiaf126 |
| 2019 | Estimation of physiological genomic estimated breeding values (PGEBV) combining full hyperspectral and marker data across environments for grain yield under combined heat and drought stress in tropical maize (Zea mays L.). | AI/prediction original research | direct | tropical maize; markers; hyperspectral bands | cross-environment validation; direct combined-stress prediction | 10.1371/journal.pone.0212200 |
| 2026 | An artificial neural network-based deep learning model to predict combined stress impact and interaction in plants. | AI/prediction original research | direct | cross-species plants; rice validation; environmental descriptors | hold-out evaluation; rice yield check; direct AI application; external biological validation remains narrow | 10.1002/aps3.70047 |
| 2023 | When drought meets heat - a plant omics perspective. | review/context | context only | See article metadata/abstract | Original study; detailed design assessed in narrative where relevant | 10.3389/fpls.2023.1250878 |

| Year | Study | Evidence class | Relationship | System / layers | Validation and interpretation | DOI |
|---|---|---|---|---|---|---|
| 2025 | A Review of Differential Plant Responses to Drought, Heat, and Combined Drought + Heat Stress. | review/context | context only | See article metadata/abstract | Original study; detailed design assessed in narrative where relevant | 10.3390/cimb47120975 |
| 2026 | AI-Augmented Multi-Omics for Abiotic Stress Responses: A New Frontier in Plant Hormone Systems Biology. | review/context | context only | See article metadata/abstract | Original study; detailed design assessed in narrative where relevant | 10.3390/plants15142185 |
| 2020 | Tolerance of Combined Drought and Heat Stress Is Associated With Transpiration Maintenance and Water Soluble Carbohydrates in Wheat Grains. | direct combined-stress original research | direct | See article metadata/abstract | Original study; detailed design assessed in narrative where relevant | 10.3389/fpls.2020.568693 |
| 2026 | Deciphering acclimation to sublethal combined and sequential abiotic stresses in Arabidopsis thaliana. | direct combined-stress original research | direct | See article metadata/abstract | Original study; detailed design assessed in narrative where relevant | 10.1093/plphys/kiae581 |
| 2025 | Transcriptional Dynamics of Rice Under Individual, Combined, and Sequential Abiotic Stresses: Insights Across Stress Severity and Recovery in Contrasting Genotypes. | direct combined-stress original research | direct | See article metadata/abstract | Original study; detailed design assessed in narrative where relevant | 10.1111/pce.70062 |
| 2017 | Relationships between drought, heat and air humidity responses revealed by transcriptome-metabolome co-analysis. | direct combined-stress original research | indirect/supporting | See article metadata/abstract | Original study; detailed design assessed in narrative where relevant | 10.1186/s12870-017-1062-y |
| 2019 | Metabolic responses of rice source and sink organs during recovery from combined drought and heat stress in the field. | direct combined-stress original research | direct | rice source and sink organs; metabolome; recovery physiology | field recovery sampling; direct recovery evidence | 10.1093/gigascience/giz102 |
| 2020 | Do plants respond and recover from a combination of drought and heatwave in the same manner under adequate and deprived soil nutrient conditions? | direct combined-stress original research | direct | See article metadata/abstract | Original study; detailed design assessed in narrative where relevant | 10.1016/j.plantsci.2019.110333 |
| 2015 | Metabolic and transcriptomic signatures of rice floral organs reveal sugar starvation as a factor in reproductive failure under heat and drought stress. | direct combined-stress original research | indirect/supporting | rice floral organs; transcriptome; metabolome | tissue-specific controlled experiment; direct reproductive multi-omics evidence | 10.1111/pce.12545 |
| 2022 | Metabolomic and transcriptomic analyses reveal that sucrose synthase regulates maize pollen viability under heat and drought stress. | direct combined-stress original research | indirect/supporting | maize pollen; transcriptome; metabolome; functional assay | molecular and functional validation; direct reproductive evidence | 10.1016/j.ecoenv.2022.114191 |
| 2023 | The transcriptome of soybean reproductive tissues subjected to water deficit, heat stress, and a combination of water deficit and heat stress. | direct combined-stress original research | direct | See article metadata/abstract | Original study; detailed design assessed in narrative where relevant | 10.1111/tpj.16222 |
| 2026 | Metabolic remodeling and sucrose transporter modulation in source-sink tissues conserve pollen | direct combined-stress original research | direct | rice source-sink tissues; metabolites; | tissue-resolved validation; direct yield-proximal evidence | 10.1016/j.plantsci.2025.112946 |

| Year | Study | Evidence class | Relationship | System / layers | Validation and interpretation | DOI |
|---|---|---|---|---|---|---|
| | germination and spikelet fertility in rice under concomitant heat and drought stress. | | | transporters; fertility traits | | |
| 2022 | Primary root response to combined drought and heat stress is regulated via salicylic acid metabolism in maize. | direct combined-stress original research | direct | See article metadata/abstract | Original study; detailed design assessed in narrative where relevant | 10.1186/s12870-022-03805-4 |
| 2022 | Genome-Wide Association Study Reveals Genetic Architecture and Candidate Genes for Yield and Related Traits under Terminal Drought, Combined Heat and Drought in Tropical Maize Germplasm. | genomics original research | direct | tropical maize; SNPs; yield traits | field multi-environment; direct combined-stress field evidence | 10.3390/genes13020349 |
| 2010 | Heat and drought adaptive QTL in a wheat population designed to minimize confounding agronomic effects. | genomics original research | indirect/supporting | wheat; markers; adaptive traits | designed population; foundational genomics evidence | 10.1007/s00122-010-1351-4 |
| 2017 | Mapping QTLs associated with agronomic and physiological traits under terminal drought and heat stress conditions in wheat (Triticum aestivum L.). | genomics original research | indirect/supporting | See article metadata/abstract | Original study; detailed design assessed in narrative where relevant | 10.1139/gen-2016-0017 |
| 2019 | Genome-wide association analyses for yield and yield-related traits in bread wheat (Triticum aestivum L.) under pre-anthesis combined heat and drought stress in field conditions. | genomics original research | direct | bread wheat; markers; yield traits | field conditions; direct field evidence | 10.1371/journal.pone.0213407 |
| 2023 | QTL mapping: insights into genomic regions governing component traits of yield under combined heat and drought stress in wheat. | genomics original research | direct | wheat; markers; component traits | field evaluation; requires fine mapping | 10.3389/fgene.2023.1282240 |
| 2025 | Genome-wide association mapping for stay-green and stem reserve mobilization traits in wheat (Triticum aestivum L.) under combined heat and drought stress. | genomics original research | direct | wheat; markers; stay-green; reserve mobilization | field combined stress; direct genomics evidence | 10.1007/s00709-025-02031-7 |
| 2019 | Novel Alleles for Combined Drought and Heat Stress Tolerance in Wheat. | genomics original research | direct | wheat; alleles; physiology; yield | field and genetic validation; strong translational evidence | 10.3389/fpls.2019.01800 |
| 2020 | Transcripts of wheat at a target locus on chromosome 6B associated with increased yield, leaf mass and chlorophyll index under combined drought and heat stress. | direct combined-stress original research | direct | See article metadata/abstract | Original study; detailed design assessed in narrative where relevant | 10.1371/journal.pone.0241966 |
| 2024 | High-throughput phenotyping using hyperspectral indicators supports the genetic dissection of yield in durum wheat grown under heat and drought stress. | direct combined-stress original research | indirect/supporting | See article metadata/abstract | Original study; detailed design assessed in narrative where relevant | 10.3389/fpls.2024.1470520 |

| Year | Study | Evidence class | Relationship | System / layers | Validation and interpretation | DOI |
|---|---|---|---|---|---|---|
| 2025 | A high-throughput phenotyping dataset for GWAS analysis of maize under combined drought and heat stress. | genomics original research | direct | maize; imaging phenotypes; markers | controlled phenotyping; dataset rather than causal validation | 10.1016/j.dib.2025.111947 |
| 2019 | Nondestructive Phenomic Tools for the Prediction of Heat and Drought Tolerance at Anthesis in Brassica Species. | AI/prediction original research | indirect/supporting | See article metadata/abstract | Original study; detailed design assessed in narrative where relevant | 10.34133/2019/3264872 |
| 2013 | Transcriptome responses to combinations of stresses in Arabidopsis. | direct combined-stress original research | direct | See article metadata/abstract | Original study; detailed design assessed in narrative where relevant | 10.1104/pp.112.210773 |
| 2014 | Transcriptomic analysis of Sorghum bicolor responding to combined heat and drought stress. | direct combined-stress original research | direct | See article metadata/abstract | Original study; detailed design assessed in narrative where relevant | 10.1186/1471-2164-15-456 |
| 2015 | Temporal transcriptome profiling reveals expression partitioning of homeologous genes contributing to heat and drought acclimation in wheat (Triticum aestivum L.). | direct combined-stress original research | indirect/supporting | See article metadata/abstract | Original study; detailed design assessed in narrative where relevant | 10.1186/s12870-015-0511-8 |
| 2018 | Transcriptome profilling analysis characterized the gene expression patterns responded to combined drought and heat stresses in soybean. | direct combined-stress original research | direct | See article metadata/abstract | Original study; detailed design assessed in narrative where relevant | 10.1016/j.compbiolchem.2018.09.012 |
| 2021 | Dissecting the molecular responses of lentil to individual and combined drought and heat stresses by comparative transcriptomic analysis. | direct combined-stress original research | direct | See article metadata/abstract | Original study; detailed design assessed in narrative where relevant | 10.1016/j.ygeno.2020.12.038 |
| 2021 | Transcriptome Analysis of Lolium temulentum Exposed to a Combination of Drought and Heat Stress. | direct combined-stress original research | direct | See article metadata/abstract | Original study; detailed design assessed in narrative where relevant | 10.3390/plants10112247 |
| 2018 | Global profiling of alternative splicing landscape responsive to drought, heat and their combination in wheat (Triticum aestivum L.). | direct combined-stress original research | direct | See article metadata/abstract | Original study; detailed design assessed in narrative where relevant | 10.1111/pbi.12822 |
| 2020 | Alternative splicing in tea plants was extensively triggered by drought, heat and their combined stresses. | direct combined-stress original research | direct | See article metadata/abstract | Original study; detailed design assessed in narrative where relevant | 10.7717/peerj.8258 |
| 2022 | [Drought and Heat Stress-Mediated Modulation of Alternative Splicing in the Genes Involved in Biosynthesis of Metabolites Related to Tea Quality]. | direct combined-stress original research | indirect/supporting | See article metadata/abstract | Original study; detailed design assessed in narrative where relevant | 10.31857/S0026898422020057 |
| 2020 | The cis-regulatory codes of response to combined heat and drought stress in Arabidopsis thaliana. | direct combined-stress original research | direct | See article metadata/abstract | Original study; detailed design assessed in narrative where relevant | 10.1093/nargab/lqaa049 |
| 2010 | Proteomic study of Carissa spinarum in response to combined heat and drought stress. | direct combined-stress original research | direct | See article metadata/abstract | Original study; detailed design assessed in narrative where relevant | 10.1002/pmic.200900637 |

| Year | Study | Evidence class | Relationship | System / layers | Validation and interpretation | DOI |
|---|---|---|---|---|---|---|
| 2013 | Leaf proteome alterations in the context of physiological and morphological responses to drought and heat stress in barley (Hordeum vulgare L.). | direct combined-stress original research | indirect/supporting | See article metadata/abstract | Original study; detailed design assessed in narrative where relevant | 10.1093/jxb/ert158 |
| 2015 | Characterization of common and distinctive adjustments of wild barley leaf proteome under drought acclimation, heat stress and their combination. | direct combined-stress original research | direct | See article metadata/abstract | Original study; detailed design assessed in narrative where relevant | 10.1007/s11103-015-0291-4 |
| 2016 | The Difference of Physiological and Proteomic Changes in Maize Leaves Adaptation to Drought, Heat, and Combined Both Stresses. | direct combined-stress original research | direct | See article metadata/abstract | Original study; detailed design assessed in narrative where relevant | 10.3389/fpls.2016.01471 |
| 2024 | Proteomic dynamics revealed sex-biased responses to combined heat-drought stress in Marchantia. | direct combined-stress original research | direct | See article metadata/abstract | Original study; detailed design assessed in narrative where relevant | 10.1111/jipb.13753 |
| 2025 | Comparative Leaf Proteome Analysis of Maize (Zea mays L.) Exposed to Combined Drought and Heat Stress. | direct combined-stress original research | direct | See article metadata/abstract | Original study; detailed design assessed in narrative where relevant | 10.3390/plants14223419 |
| 2026 | Stress-Specific Carbonylation and Proteasome 20S Activity in Potato Under Drought, Elevated Temperature, and Combined Stresses: Linking Oxidative Damage to Proteome Regulation. | direct combined-stress original research | direct | See article metadata/abstract | Original study; detailed design assessed in narrative where relevant | 10.3390/plants15060939 |
| 2018 | Dynamics of metabolic responses to periods of combined heat and drought in Arabidopsis thaliana under ambient and elevated atmospheric CO2. | direct combined-stress original research | direct | See article metadata/abstract | Original study; detailed design assessed in narrative where relevant | 10.1093/jxb/ery055 |
| 2016 | Differential physiological response of the grapevine varieties Touriga Nacional and Trincadeira to combined heat, drought and light stresses. | direct combined-stress original research | direct | See article metadata/abstract | Original study; detailed design assessed in narrative where relevant | 10.1111/plb.12410 |
| 2016 | Activation of Secondary Metabolism in Citrus Plants Is Associated to Sensitivity to Combined Drought and High Temperatures. | direct combined-stress original research | direct | See article metadata/abstract | Original study; detailed design assessed in narrative where relevant | 10.3389/fpls.2016.01954 |
| 2021 | Assessing Drought and Heat Stress-Induced Changes in the Cotton Leaf Metabolome and Their Relationship With Hyperspectral Reflectance. | direct combined-stress original research | indirect/supporting | See article metadata/abstract | Original study; detailed design assessed in narrative where relevant | 10.3389/fpls.2021.751868 |
| 2023 | Trichoderma spp.-mediated mitigation of heat, drought, and their combination on the Arabidopsis thaliana holobiont: a metabolomics and metabarcoding approach. | direct combined-stress original research | direct | See article metadata/abstract | Original study; detailed design assessed in narrative where relevant | 10.3389/fpls.2023.1190304 |

| Year | Study | Evidence class | Relationship | System / layers | Validation and interpretation | DOI |
|---|---|---|---|---|---|---|
| 2024 | A multi-omics approach to unravel the interaction between heat and drought stress in the Arabidopsis thaliana holobiont. | direct combined-stress original research | indirect/supporting | Arabidopsis holobiont; metabolomics; metabarcoding; physiology | factorial controlled experiment; direct multi-omics evidence | 10.3389/fpls.2024.1484251 |
| 2024 | Genome-wide profiling of histone (H3) lysine 4 (K4) tri-methylation (me3) under drought, heat, and combined stresses in switchgrass. | direct combined-stress original research | direct | switchgrass; H3K4me3; transcriptome | factorial controlled experiment; direct chromatin evidence | 10.1186/s12864-024-10068-w |
| 2026 | DNA Methylation-Mediated Reprogramming of Gene Expression and Flowering Under Combined Heat and Drought Stress in Maize. | direct combined-stress original research | direct | maize; DNA methylation; transcriptome; flowering | controlled experiment; direct epigenomic evidence | 10.1111/pce.70654 |
| 2025 | Mapping stress memory: genetic and epigenetic insights into combined drought and heat tolerance in barley. | direct combined-stress original research | direct | See article metadata/abstract | Original study; detailed design assessed in narrative where relevant | 10.1007/s00299-025-03501-1 |
| 2021 | Combined drought and heat stresses trigger different sets of miRNAs in contrasting potato cultivars. | direct combined-stress original research | direct | See article metadata/abstract | Original study; detailed design assessed in narrative where relevant | 10.1007/s10142-021-00793-w |
| 2023 | Predicting transcriptional responses to heat and drought stress from genomic features using a machine learning approach in rice. | AI/prediction original research | indirect/supporting | rice; promoter/genomic features; transcript responses | cross-validation; single-stress prediction; not causal | 10.3389/fpls.2023.1212073 |
| 2023 | Interpretable machine learning-accelerated seed treatment using nanomaterials for environmental stress alleviation. | AI/prediction original research | indirect/supporting | multiple crops; material descriptors; stress phenotypes | cross-validation plus experiments; indirect to combined drought-heat | 10.1039/d3nr02322b |
| 2026 | Genomic and Physiological Insights Into Heat-Drought Tolerance in Wheat Through GWAS and Phenotypic Evaluation. | genomics original research | indirect/supporting | wheat; SNPs; physiology; yield | field phenotyping; direct genomics evidence | 10.1111/pce.70510 |
| 2026 | Physiological and molecular response of soybean to combined drought and heat stresses during flowering. | direct combined-stress original research | direct | See article metadata/abstract | Original study; detailed design assessed in narrative where relevant | 10.1186/s12870-025-08081-6 |
| 2026 | Transcriptome-based investigation of the response and repair mechanisms in the photosynthetic system of Cycas panzhihuaensis under dual high-temperature and drought stress. | direct combined-stress original research | indirect/supporting | See article metadata/abstract | Original study; detailed design assessed in narrative where relevant | 10.1186/s12870-026-08554-2 |

| Year | Study | Evidence class | Relationship | System / layers | Validation and interpretation | DOI |
|---|---|---|---|---|---|---|
| 2025 | Proteomic dataset of sorghum leaf and root responses to single and combined drought and heat stress. | direct combined-stress original research | direct | See article metadata/abstract | Original study; detailed design assessed in narrative where relevant | 10.1038/s41597-025-04369-2 |
| 2025 | Stage-specific transcriptomic responses of wheat to heat, drought, and combined stress: AP2/ERF regulatory networks and functional characterization of TaEREBP1-L. | direct combined-stress original research | direct | See article metadata/abstract | Original study; detailed design assessed in narrative where relevant | 10.1186/s12870-025-07615-2 |
| 2025 | Synergistic Regulation at Physiological, Transcriptional, and Metabolic Levels in Dendrobium huoshanense Plants Under Combined Drought and High-Temperature Stress. | direct combined-stress original research | direct | See article metadata/abstract | Original study; detailed design assessed in narrative where relevant | 10.3390/genes16030287 |
| 2023 | Omics analyses in citrus reveal a possible role of RNA translation pathways and Unfolded Protein Response regulators in the tolerance to combined drought, high irradiance, and heat stress. | direct combined-stress original research | direct | See article metadata/abstract | Original study; detailed design assessed in narrative where relevant | 10.1093/hr/uhad107 |
| 2022 | Elevated CO2 Improves the Physiology but Not the Final Yield in Spring Wheat Genotypes Subjected to Heat and Drought Stress During Anthesis. | direct combined-stress original research | indirect/supporting | See article metadata/abstract | Original study; detailed design assessed in narrative where relevant | 10.3389/fpls.2022.824476 |
| 2022 | Multiple Abiotic Stresses Applied Simultaneously Elicit Distinct Responses in Two Contrasting Rice Cultivars. | direct combined-stress original research | indirect/supporting | See article metadata/abstract | Original study; detailed design assessed in narrative where relevant | 10.3390/ijms23031739 |
| 2022 | Reduction of heat stress pressure and activation of photosystem II repairing system are crucial for citrus tolerance to multiple abiotic stress combination. | direct combined-stress original research | direct | See article metadata/abstract | Original study; detailed design assessed in narrative where relevant | 10.1111/ppl.13809 |
| 2021 | Exploitation of Tolerance of Wheat Kernel Weight and Shape-Related Traits from Aegilops tauschii under Heat and Combined Heat-Drought Stresses. | direct combined-stress original research | direct | See article metadata/abstract | Original study; detailed design assessed in narrative where relevant | 10.3390/ijms22041830 |
| 2019 | Phenotypic and metabolic plasticity shapes life-history strategies under combinations of abiotic stresses. | direct combined-stress original research | direct | See article metadata/abstract | Original study; detailed design assessed in narrative where relevant | 10.1002/pld3.113 |
| 2017 | Detecting early signs of heat and drought stress in Phoenix dactylifera (date palm). | direct combined-stress original research | indirect/supporting | See article metadata/abstract | Original study; detailed design assessed in narrative where relevant | 10.1371/journal.pone.0177883 |
| 2016 | Expression of OsMYB55 in maize activates stress-responsive genes and enhances heat and drought tolerance. | direct combined-stress original research | indirect/supporting | See article metadata/abstract | Original study; detailed design assessed in narrative where relevant | 10.1186/s12864-016-2659-5 |

| Year | Study | Evidence class | Relationship | System / layers | Validation and interpretation | DOI |
|---|---|---|---|---|---|---|
| 2013 | Simultaneous application of heat, drought, and virus to Arabidopsis plants reveals significant shifts in signaling networks. | direct combined-stress original research | indirect/supporting | See article metadata/abstract | Original study; detailed design assessed in narrative where relevant | 10.1104/pp.113.221044 |
| 2008 | Ascorbate peroxidase 1 plays a key role in the response of Arabidopsis thaliana to stress combination. | direct combined-stress original research | direct | See article metadata/abstract | Original study; detailed design assessed in narrative where relevant | 10.1074/jbc.M806337200 |
